# Alteration of Tight and Adherens Junctions on 50-Hz Magnetic Field Exposure in Madin Darby Canine Kidney (MDCK) Cells

**DOI:** 10.1100/tsw.2004.181

**Published:** 2004-10-20

**Authors:** Zoltán Somosy, Zsolt Forgács, Gabriella Bognár, Katalin Horváth, Gyöző Horváth

**Affiliations:** “Fodor József” National Center of Public Health, “Frédéric Joliot-Curie” National Research Institute for Radiobiology and Radiohygiene, Budapest, Hungary; ^2^ “Fodor József” National Center of Public Health, National Institute of Chemical Safety, Budapest, Hungary; ^3^ Institute of Health Protection, Hungarian Defence Forces, Budapest, Hungary

**Keywords:** adherens junction, tight junction, magnetic field, occludin, cadherin, β-catenin, Wnt signal, immunohistochemistry

## Abstract

Adherens (AJ) and tight junctions (TJ), as integrated parts of the junctional complex, are multifunctional specialized regions of the cell membrane in epithelial cells. They are responsible for cell-to-cell interactions and also have great importance in cellular signaling processes including Wnt protein-mediated signals. As electromagnetic field (EMF) exposure is known to cause alterations in the function as well as supramolecular organization of different cell contacts, our goal was to investigate the effect of 50-Hz magnetic field (MF) exposures on the subcellular distribution of some representative structural proteins (occludin, β-catenin, and cadherin) found in AJ and TJ. Additionally, cellular β-catenin content was also quantified by Western blot analysis. 50-Hz MF exposures seemed to increase the staining intensity (amount) of occludin, cadherins, and β-catenin in the junctional area of MDCK cells, while Western blot data indicated the quantity of b-catenin was found significantly decreased at both time points after EM exposures. Our results demonstrate that MF are able to modify the distribution of TJ and AJ structural proteins, tending to stabilize these cell contacts. The quantitative changes of β-catenin suggest a causative relationship between MF effects on the cell junctional complex and the Wnt signaling pathway.

### INTRODUCTION

Cell contacts are multifunctional specialized regions of the cell membrane. They are responsible for establishing cell-to-cell and cell-to-matrix physical contacts and also play critical roles in epithelial barrier functions, intercellular communication mechanisms, as well as biological processes that are involved in or related to cell signaling[1,2,3,4]. Tight (TJ) and adherens junctions (AJ) are stable and sophisticated molecular complexes establishing multifunctional connections between the junctional complexes of epithelial cells. These cell couplings physically stabilize the neighboring cells, regulate the paracellular transports of ions and other matters, inhibit the lateral shift of membrane proteins, and their signal transmitter and signal generating function is also known[1,2,3,4,5]. The respective coupling proteins in TJ and AJ are the claudins, occludin, and cadherins, while in AJ ZO proteins and catenins are the linkers to the cytoskeleton[1,2,3,4,5]. Recently, it has been recognized that ZO-1 protein is a common structural protein for both TJ and AJ as ZO-1 is able to bind to many proteins, including ZO-2 and ZO-3, occludin, claudins, ZO-1–associated nucleic acid–binding protein, and α-catenin[[6,7]. This ZO-1–mediated connection is the structural basis of the cooperation between AJ and TJ.

Beyond its structural role in AJ, ß-catenin also has an important second function, i.e., in its unbound free form it can act as a transcriptional cofactor when stimulated by the Wnt signal transduction pathway[2,4,5,8,9,10,11]. This signaling pathway is involved in a variety of cellular processes including cell proliferation, differentiation, embryogenesis, and oncogenesis. In this process, cells can transmit information from the cell surface to the nucleus. In Wnt signaling, the presence and amount of ß-catenin is regulated by the ratio of its free and bound forms, and also by the amount of the free protein in the cytoplasmic pool[8,9,10,11].

Biological membranes, including cell membrane, are playing an essential role in the cellular responses to electric and electromagnetic field (EMF) exposures[12,13,14]. However, the mechanism(s) standing behind the biophysical interactions at the cell membrane are still not exactly understood. It is generally accepted that the initial biochemical or metabolic state of the cell membrane, as well as the functional and supramolecular organization of its specific regions (receptors, cell contact areas), are sensitive targets of electric and EMF exposures[15,16,17,18,19]. Following EMF exposures, structural and functional changes are detectable in various types of cell contact sites[20,21,22,23,24,25,26,27]. There are available data describing how gap junction–mediated intercellular chemical communications are modified by EMF exposures[20,21]. The EMF-induced expression and/or redistribution of certain integrins and other adhesion molecules can modify cell-to-cell and cell-to-extracellular matrix (ECM) contacts[22,23,24,25,26,27].

Nowadays, studies on the TJ- and AJ-mediated blood-brain barrier alterations following microwave and MF exposures are favored areas of nonionizing cellular radiobiology. By now it has become evident that this barrier is highly sensitive to the heat generated by microwaves in the exposed biological target. On the other hand, there are some conflicting data regarding the involvement of the nonthermal effects of microwave and MF exposures in these processes[28,29,30]. As AJ and TJ are involved in the regulation of this permeability barrier, it is important to know whether the function, biochemical characteristics, localization, and quantity of their structural proteins are altered on nonionizing field exposure. Though the nonionizing radiation–induced redistribution of junctional proteins is known from the literature, this effect has not been investigated so far on *in vitro* cellular model systems generally used for junctional complex studies.

Therefore, in this work, the effect of 50-Hz EMF exposure was studied on the localization characteristics of occludin, cadherins, and β-catenin in such a cellular model, i.e., Madin-Darby canine kidney (MDCK) cells by immunohistochemistry. These investigations inform about the MF-induced structural changes of the junctional strands, as well as about the relationship between the quantity and staining characteristics of junctional proteins. Taking into account that the so-called transcriptional cofactor function of ß-catenin highly depends on its total cellular concentration, Western blotting for this protein was also included in our methodology.

## MATERIALS AND METHODS

### Cell Culture

Madin-Darby canine kidney cells (MDCK cells) were plated in 24-well polystyrene culture plates on glass coverslips and maintained in Dulbecco's Modified Eagle's Medium (containing 10% fetal calf serum, 4 m*M* L-glutamine, 10 units/ml penicillin, and 10 mg/ml streptomycin) at 37°C in a 5% $CO_2$ moist atmosphere. For studies, confluent cultures of MDCK cells were used.

### AC MF Exposures

Cell cultures were exposed in a $CO_2$ incubator at 37°C to AC MF vertically parallel to the cylinder axis of a cylindrical recording chamber. The frequency of the field was 50 Hz; the flux density B (AC) was 500 μT (root mean square). Two coils in the Helmholtz arrangement were used to produce the vertical AC MF. Coils were manufactured from 0.3-mm copper wire with an inner diameter of 12 cm and using 85 turns for each coil. They were connected collaterally (as a pair) with the resultant resistance of 4.2 ohms. The MF was measured by a three-dimensional MF meter (Fluxset 3C), i.e., a one-dimensional Hall-probe connected to a gaussmeter (EFA-3) MF measurement system. The spectral component of the applied and background MF was also analyzed. In the test laboratory, Earth's MF was 46 μT. The background 50-Hz MF in the incubator was 40/300 nT (heating off/on). The stray field of the applied 50-Hz MF at the place of the unexposed control was 1–4 μT, whereas the first (100 Hz) and second (150 Hz) harmonics were less than 8 and 3%, respectively. The AC MF was generated by a power audio complex generator. Cells were fixed for immunocytochemistry after 4 and 24 h of exposure to the MF.

### Immunohistochemistry

For the immunohistochemical detection of occludin, cadherins, and β catenin, cells were fixed and permeabilized with methanol at –20°C for at least 2 h, 4–24 h after MF exposures. The monoclonal antipancadherin (Clone CH-19) and monoclonal anti-β-catenin (Clone 15B8) obtained from Sigma Company (St. Louis, MO, USA) were used in respective dilutions of 1:300 and 1:1000 at room temperature for 1 hr. For the detection of occludin, antioccludin antibody (Clone no. OC-3F10) obtained from Zymed Laboratories (San Francisco, CA, USA) was used in a 1:300 dilution at room temperature. The FITC-labeled antimouse secondary antibody (developed in rabbits) was obtained from Sigma Company. Cells were then mounted in Vectashield mounting medium (Vector Lab. Inst. Burgilame, CA, USA) and investigated in Axioskope (Zeiss, Germany) fluorescent microscope. Pictures were taken on color slides (Fujichrome Sensia, ISO 100) and scanned in by a slide scanner (Minolta Dimage Scan Elite II ) using 2820 dpi optical resolution, and printed by a Hewlett Packard DeskJet 990C printer.

### Protein Isolation and Western Blotting

Western blotting technique was used to detect and measure the relative amount of β-catenin expressed in the control and MF-exposed (500 μT, 4 and 24 h) MDCK cells.

Cell monolayers from TC 80 flasks were washed twice with PBS and then rubbed off in 2-ml PBS with rubber police. Cell suspensions were centrifuged at 1500*g* for 8 min. After removing the supernatant, the residue was lysed in TRIS-EDTA buffer (0.02 *M* Tris, 0.001 *M* EDTA) containing 1% dithiotreitol (1 *M* DTT) and 0.1% phenylmethylsulfonyl fluoride (0.1 *M* PMSF). Aliquots of the cell lysates were used for the evaluation of the protein content by Bradford assay. Protein samples (20 μg of total protein/lane)

were separated by electrophoresis for 45 min at 200 V using 7% sodium dodecyl sulphate polyacrylamide gel electrophoresis (SDS-PAGE) gels.

Protein samples were transferred to polyvinyldiene difluoride (PVDF) membranes (Hybond-P, Amersham, England) by electroblotting at 4°C for 2 h at 100 V. In case of repeated use, membranes were stripped in Tris-HCl buffer containing 2% SDS and 0.1 *M* β-mercaptoethanol for 30 min at 50°C, and then stored at –20°C until use. Membranes were placed in polyethylene bags and blocked for 1 h at room temperature in 5% fat-free milk diluted in Tris-buffered saline (TBS: 10 m*M* Tris-HCl and 150 m*M* NaCl pH:7,4) and containing 0.1% TritonX-100 and stored at –20°C until use. The blots were washed in TBS-T (0.1% TritonX-100 diluted in TBS). The washing buffer was replaced with the monoclonal (mouse) primary antibody diluted 1:500 in TBS-T buffer. The blots were incubated at room temperature for 2 h or, in the case of stripped membranes, overnight with the β-catenin (SIGMA, St. Louis, MO, USA) and pan-cadherin antibodies (SIGMA, St. Louis, MO, USA). The blots were then washed in TBS-T and incubated in horseradish peroxidase–linked antimouse IgG (Amersham, Oakville, ON, Canada) as a secondary antibody diluted 1:2500 in TBS-T for 1.5 h at room temperature. After repeated washing in TBS-T, antibody-antigen complexes in all membranes were detected by chemiluminescence (ECL kit, Amersham Life Science, Oakville, ON, Canada) and exposed to photographic film (FORTEPAN 200, Hungary) for 30 min. The Adobe PhotoDeluxe 1.0 (Adobe Systems, Inc.) was used to scan the protein bands and these were quantified using the 1D image analysis software (Version 3.5).

### RESULTS

Cultured MDCK cells are polarized and have a well-developed junctional complex consisting of TJ and AJ[31]. In control cells, the occludin (Fig. 1A), pan-cadherin (Fig. 1E), and β-catenin staining (Fig. 1C) distribute mainly along the borders of the cell membrane appearing as a more-or-less continuous line and show characteristic honeycomb-like pattern at the zone of the junctional complex. Accumulation of cadherins and β-catenin is also observable at the basal region of the cells (Figs. 1C and 1E) while a faint diffuse staining pattern is characteristic to the cytoplasm. Rounded (probably mitotic) cells with intensive staining in their apical region also appear in the cultures (Figs. 1C–E).

This characteristic staining pattern of the cells and cell contact regions was not altered by the MF neither 4 nor 24 h after the exposure. However, the overall staining intensity of both antibodies significantly increased in the cell contact areas (Fig. 1B, D, F).

According to our Western blot data, the quantity of β-catenin was found significantly decreased at both time points after MF exposures (Fig. 2).

### DISCUSSION

Our presented results indicate that MF exposure increased the staining intensity of all investigated junctional proteins (occludin, cadherin, and β-catenin) at the cell border region, i.e., at the junctional areas of MDCK cells. It is known and generally accepted in the literature that in epithelial and endothelial cells, including MDCK cells, a good correlation exist between the staining intensity, pattern, and the actual organization and amount of junctional proteins of TJ and AJ. Different physical and chemical agents, toxic metal exposure, ionizing irradiation, for example, lead to the disorganization or fragmentation as well as destabilization of tight junctional strands, and also resulted in the decreased staining of occludin and other structural proteins of TJ (ZO-1)[32,33,34,35,36,37,38,39]. On the other hand, from the accumulation of the structural proteins (indicated by the increased staining intensity) in the region of the junctional complex, one can conclude the stabilization of cell contacts. Based on our results, MF exposures are supposed to strengthen or stabilize the structural integrity of TJ and AJ. These results are entirely coherent with our earlier data obtained by microwave exposures. Carrying out detailed morphometric analysis on tight junctional strands after microwave *in vivo* exposure of mouse jejunal cells on freeze-fractured replicas

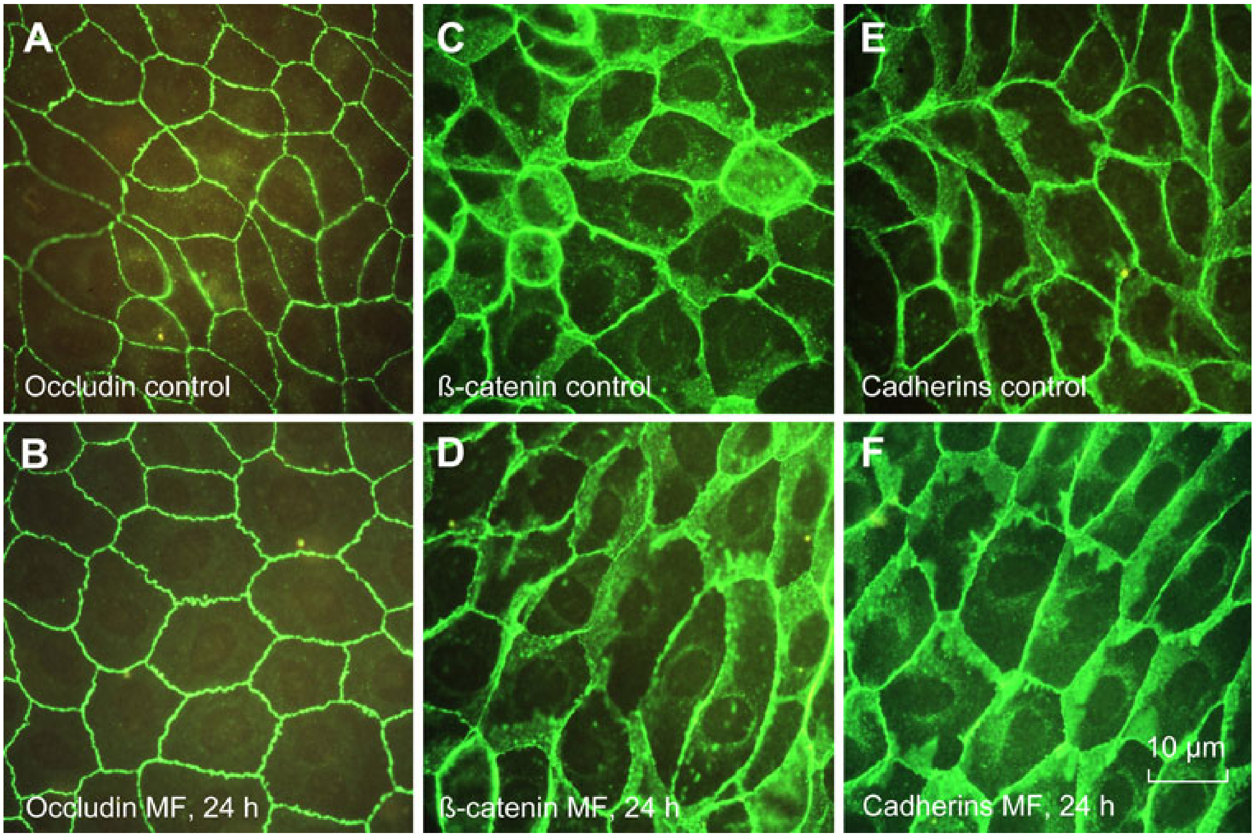

**FIGURE 1**. Cellular distribution of occludin (A), β-catenin (C), and cadherins (E) in control and in MF-exposed (50 Hz, 24 h) (B, D, and E) MDCK cells.

revealed a decrease of the so-called break index indicating the enhanced structural integrity of TJ[34]. It was reported that MF also increase the staining (the amount) intensity of specific proteins at the cell-to-cell contact area in keratinocytes[22,24], gonadal cells[26,27], leukocytes[23], and colon tumor model[25].

The fact that both TJ and AJ are somehow altered suggests that in epithelial cells the whole junctional complex responds to or affected by the EMF exposure.

TJ and AJ are dynamic structures and are regulated by different biologically active extracellular molecules (i.e., hormones, growth factors) and cellular signal transduction systems (i.e., phospholipase C, protein kinases). Second messengers of signal transduction processes, such as $Ca^{2+}$, cAMP, cGMP, and nitric oxide (NO) seem to play decisive role in this regulation both in coordinating and subordinating relations[2,3,5]. Considering that MF can alter the local calcium concentration[40,41], cAMP[16,42], and NO levels[43,44], their pathogenetic role in the junctional responses is also supposed.

Nevertheless, the functional consequence of such increase in the occludin, cadherin, or β-catenin staining, as well as the possible stabilization of cell contacts, is still not clear.

Our study shows the total amount, as well as the free cytoplasmic β-catenin significantly decreased on MF exposure. The altered concentration of free cytoplasmic β-catenin protein related to Wnt signal changes[2,4,8,10,11].

It is known that the level of ß-catenin pool is depend on the physiological state of cells[11] and it frequently decreases on different chemical and physical toxic effects, i.e., toxic metal exposures[45], ionizing irradiation[32,46,47]. Moreover, the elevated level of cytoplasmic ß-catenin is characteristic in different tumor cells[48,49]. However, at the current stage of research the behavior of free ß-catenin is not known on MF exposure.

Our presented new experimental data demonstrate how MF affect TJ and AJ. Based on these results, the possible causative relationship between MF effects and Wnt signaling pathway was first associated.

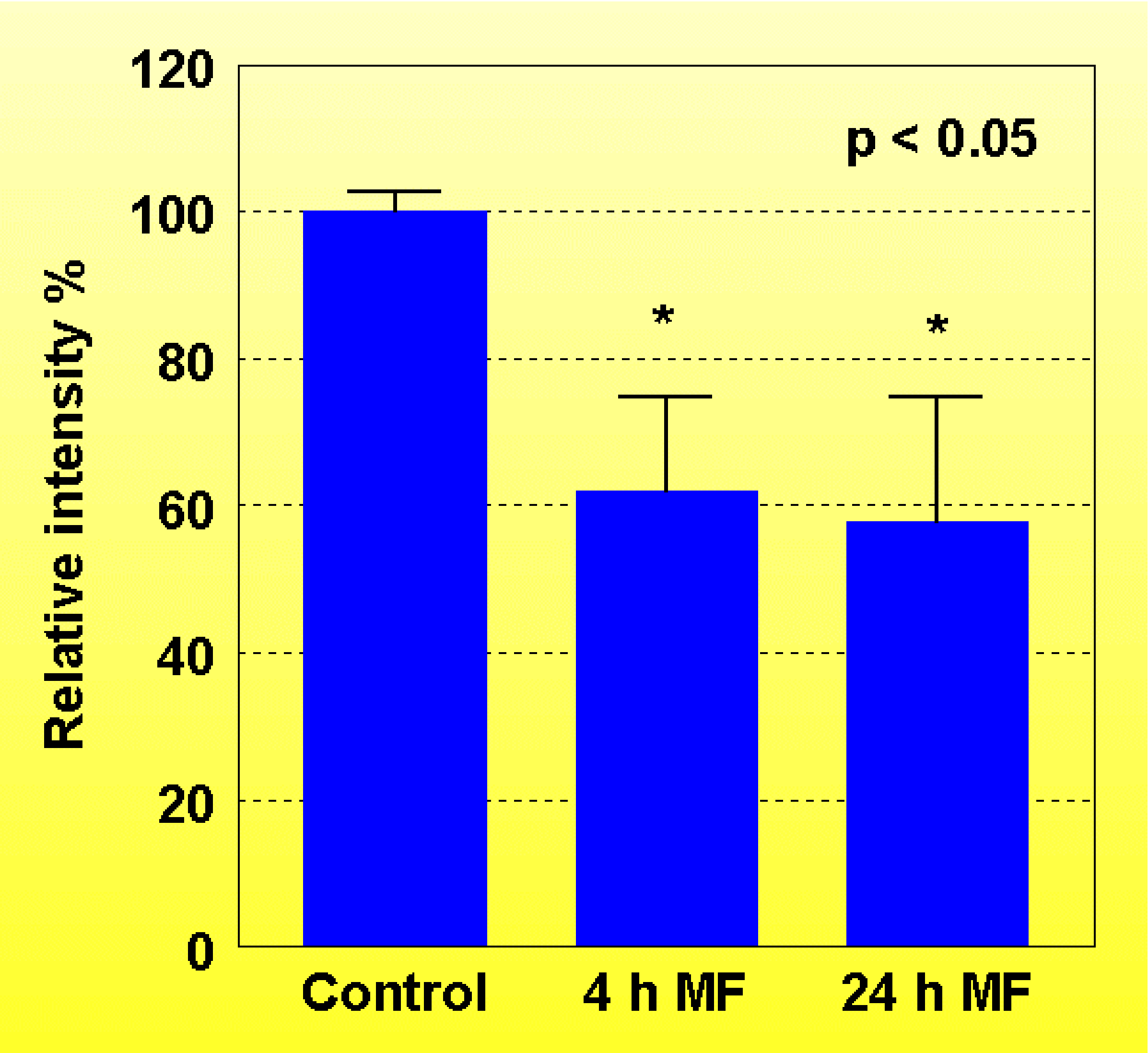

**FIGURE 2**. The relative amounts of β-catenin in the control and MF-exposed cells.

### ACKNOWLEDGMENT

Thanks are expressed to Erzsébet Fodor for her excellent technical assistance. This work was supported by the Committee for Health Studies Grant (ETT) of the Hungarian Ministry of Health, Social and Family Affairs under the contract of No. 160/2003, and by research grants from the National Scientific Research Fund (OTKA No. T 034957).
